# Supplementary material for: Immunotherapy in Hepatocellular Cancer Patients with Mild to Severe Liver Dysfunction: Adjunctive Role of the ALBI Grade
Source: Cancers (Basel). 2020 Jul 10;12(7):1862. doi: 10.3390/cancers12071862 (PMC7408648; doi:10.3390/cancers12071862)
Supplement: Supplementary file 1 [file cancers-12-01862-s001.pdf]

Supplementary Materials

Immunotherapy in Hepatocellular Cancer Patients with Mild to Severe Liver Dysfunction: Adjunctive Role of the ALBI Grade

David J. Pinato, Takahiro Kaneko, Anwaar Saeed, Tiziana Pressiani, Ahmed Kaseb, Yinghong Wang, David Szafron, Tomi Jun, Sirish Dharmapuri, Abdul Rafeh Naqash, Mahvish Muzaffar, Musharraf Navaid, Chieh-Ju Lee, Anushi Bulumulle, Bo Yu, Sonal Paul, Neil Nimkar, Dominik Bettinger, Hannah Hildebrand, Yehia I. Abugabal, Celina Ang, Thomas U. Marron, Uqba Khan, Nicola Personeni, Lorenza Rimassa and Yi-Hsiang Huang

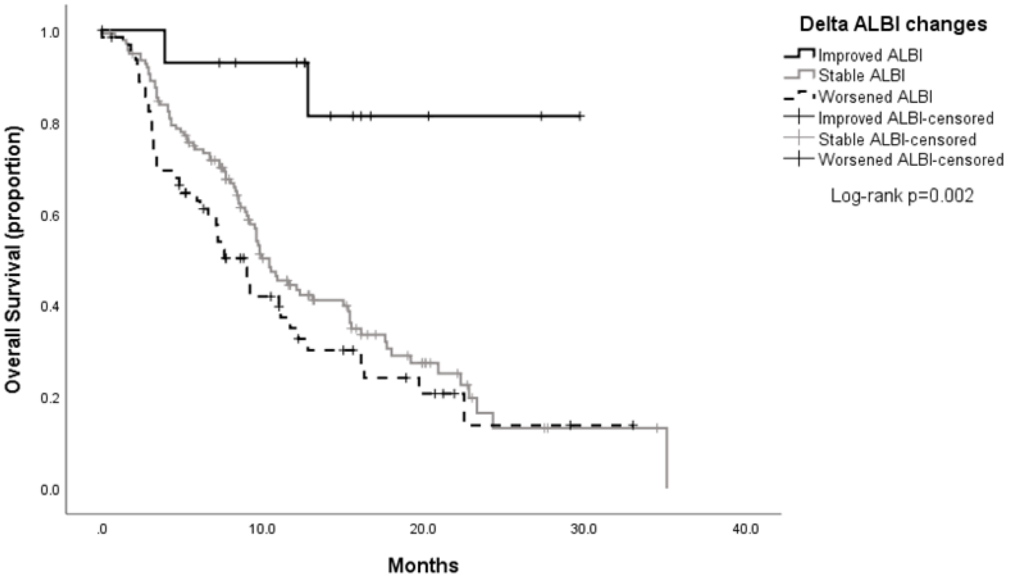

Figure S1. Dynamic changes in the ALBI grade from ICI commencement to discontinuation associated with improved OS in HCC patients.

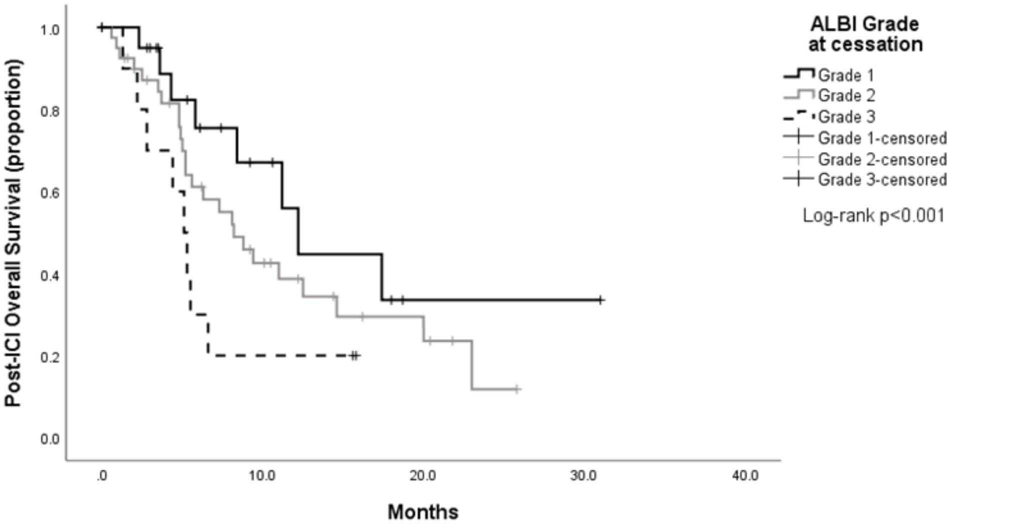

Figure S2. The relationship between ALBI grade at discontinuation and PIOS within patients in CTP A functional class at ICI discontinuation.

Table S1. Distribution of Treatment-related adverse events (trAEs) according to Child Turcotte-Pugh (CTP) classification.

trAE Class	trAE of Any Grade		
	CTP A	CTP B	CTP C
Dermatologic Toxicity	32 (10)	6 (2)	1 (0.3)
Diarrhoea/Colitis	23 (7)	2 (0.5)	0 (0)
Fatigue	47 (14)	10 (3)	0 (0)
Liver Toxicity	53 (16)	6 (2)	0 (0)
Endocrine Toxicity	20 (6)	2 (0.5)	0 (0)
Pneumotoxicity	12 (4)	1 (0.3)	0 (0)
Others	38 (12)	2 (0.5)	1 (0.3)

Table S2. Distribution of Treatment-related adverse events (trAEs) according to Albumin-bilirubin (ALBI) grade.

trAE Class	trAE of Any Grade		
	ALBI 1	ALBI 2	ALBI 3
Dermatologic Toxicity	19 (6)	12 (4)	5 (0.3)
Diarrhoea/Colitis	13 (4)	11 (3)	0 (0.3)
Fatigue	23 (7)	25 (7)	3 (0.3)
Liver toxicity	27 (8)	31 (9)	1 (0.3)
Endocrine toxicity	10 (3)	11 (3)	0 (0.3)
Pneumotoxicity	6 (2)	6 (2)	1 (0.3)
Others	33 (10)	18 (5)	1 (0.3)

Table S3. The relationship between CTP class and trAEs in patients treated with ICI as monotherapy or combinations.

ICPI Regimen	Child-Turcotte Pugh Class		No TrAE	TrAE Any grade	Total	Pearson X^2 (p Value)
Monotherapy	CTP A	N	99	75	174	1.83 (p = 0.39)
		%	57%	43%	100%	
	CTP B	N	40	21	61	
		%	66%	34%	100%	
	CTP C	N	5	2	7	
		%	72%	28%	100%	
	Total	N	144	98	242	
		%	60%	40%	100%	
Combination Therapy	CTP A	N	12	36	48	2.46 (p = 0.11)
		%	25%	75%	100%	
	CTP B	N	2	1	3	
		%	66%	34%	100%	
	Total	N	14	37	51	
		%	2%	72%	100%	
Total	CTP A	N	111	111	222	5.76 (p = 0.06)
		%	50%	50%	100%	
	CTP B	N	42	22	64	
		%	66%	34%	100%	
	CTP C	N	5	2	7	
		%	71%	29%	100%	
	Total	N	158	135	293	
		%	54%	46%	100%	

Table S4. The relationship between ALBI grade and trAEs in patients treated with ICI as monotherapy or combinations.

ICPI Regimen	ALBI Grade		No TrAE	TrAE Any grade	Total	Pearson X^2 (*p* Value)
Monotherapy	ALBI 1	N	35	34	69	4.11 (*p* = 0.12)
		%	51%	49%	100%	
	ALBI 2	N	86	54	140	
		%	61%	39%	100%	
	ALBI 3	N	22	9	31	
		%	71%	29%	100%	
	Total	N	143	97	240	
		%	60%	40%	100%	
Combination Therapy	ALBI 1	N	6	19	25	0.67 (*p* = 0.41)
		%	24%	76%	100%	
	ALBI 2	N	8	15	23	
		%	35%	65%	100%	
	Total	N	14	34	48	
		%	30%	70%	100%	
Total	ALBI 1	N	41	53	94	8.54 (*p* = 0.01)
		%	44%	56%	100%	
	ALBI 2	N	94	69	163	
		%	58%	42%	100%	
	ALBI 3	N	22	9	31	
		%	71%	29%	100%	
	Total	N	157	131	288	
		%	54%	46%	100%	

© 2020 by the authors. Licensee MDPI, Basel, Switzerland. This article is an open access article distributed under the terms and conditions of the Creative Commons Attribution (CC BY) license (http://creativecommons.org/licenses/by/4.0/).
